# XBP1s-EDEM2 Prevents the Onset and Development of HFpEF by Ameliorating Cardiac Lipotoxicity

**DOI:** 10.1161/CIRCULATIONAHA.124.072194

**Published:** 2025-03-25

**Authors:** Oveena Fonseka, Rida Raja, Claire Ross, Sanskruti R. Gare, Jiayan Zhang, Susanne S. Hille, Katharine King, Andrea Ruiz-Velasco, Namrita Kaur, Xinyi Chen, Jessica M. Miller, Riham R.E. Abouleisa, Qinghui Ou, Zhiyong Zou, Xiangjun Zhao, Cristian Sotomayor-Flores, Derk Frank, Eileithyia Swanton, Martin R. Pool, Sara Missaglia, Daniela Tavian, Gabriele G. Schiattarella, Tao Wang, Luigi Venetucci, Christian Pinali, Martin K. Rutter, Bernard D. Keavney, Elizabeth J. Cartwright, Tamer M.A. Mohamed, Oliver J. Müller, Wei Liu

**Affiliations:** Faculty of Biology, Medicine and Health, The University of Manchester, UK (O.F., R.R., C.R., S.R.G., J.Z., K.K., A.R.-V., N.K., X.C., Z.Z., X.Z., E.S., M.R.P., T.W., L.V., C.P., M.K.R., B.D.K., E.J.C., T.M.A.M., W.L.).; Departments of Internal Medicine V (S.S.H., D.F., O.J.M.), University of Kiel, Germany.; Internal Medicine III (D.F.), University of Kiel, Germany.; DZHK, German Center for Cardiovascular Research, Partner Site Hamburg/Kiel/Lübeck, Kiel, Germany (S.S.H., O.J.M.).; Surgery Department, Baylor College of Medicine, Houston, TX (J.M.M., R.R.E.A., T.M.A.M.).; Institute of Molecular Cardiology, University of Louisville, KY (Q.O., T.M.A.M.).; Max Rubner Center for Cardiovascular Metabolic Renal Research, Deutsches Herzzentrum der Charité, Charité-Universitätsmedizin Berlin, Germany (C.S.-F., G.G.S.).; DZHK, German Centre for Cardiovascular Research, Partner Site Berlin, Germany (C.S.-F., G.G.S.).; Laboratory of Cellular Biochemistry and Molecular Biology, Università Cattolica del Sacro Cuore, Milan, Italy (S.M., D.T.).; Translation Approaches in Heart Failure and Cardiometabolic Disease, Max Delbrück Center for Molecular Medicine in the Helmholtz Association, Berlin, Germany (G.G.S.).; Diabetes, Endocrinology and Metabolism Centre, NIHR Manchester Biomedical Research Centre (M.K.R.), Manchester University Hospitals NHS Foundation Trust, Manchester Academic Health Science Centre, UK.; Manchester Heart Centre (B.D.K.), Manchester University Hospitals NHS Foundation Trust, Manchester Academic Health Science Centre, UK.

**Keywords:** cardiac lipotoxicity, EDEM2, heart failure, HFpEF, metabolic stress, XBP1s

## Abstract

**BACKGROUND:**

Morbidity and mortality of heart failure with preserved ejection fraction (HFpEF) is increased in metabolic disorders. However, options for preventing and treating these prevalent outcomes are limited. Intramyocardial lipotoxicity contributes to cardiac dysfunction. Here, we investigate the mechanisms underlying EDEM2 (endoplasmic reticulum degradation–enhancing alpha-mannosidase–like protein 2) regulation of cardiac lipid homeostasis and assess strategies that inhibit the incidence and progression of HFpEF.

**METHODS:**

Metabolic stress was induced in C57BL/6 male mice using a high-fat diet and *N*ω-nitro-L-arginine methyl ester. The recombinant adeno-associated virus 9 delivery system was used for loss- and gain-of-function studies. Palmitic acid and oleic acid stimulation of rat cardiomyocytes and human induced pluripotent stem cell–derived cardiomyocytes imitated a condition of high lipids in vitro. Molecular mechanisms were investigated via RNA sequencing, mass spectrometry proteomics, lipidomic analyses, transmission electron microscopy, histology, and luciferase reporter assays.

**RESULTS:**

In the human heart, we first detected lipid overload accompanied by a reduction of *XBP1* (X-box binding protein 1) under metabolic stress. Thereafter, a decrease in EDEM2 was confirmed in human and mouse HFpEF hearts. Given that XBP1s (spliced X-box binding protein 1) is a transcription factor, EDEM2 was identified as its new target in cardiomyocytes. EDEM2 knockdown mice manifested lipid droplet accumulation and higher levels of triglycerides and diglycerides in the myocardium, aggravating oxidative stress, hypertrophy, and the onset and progression of HFpEF under metabolic stress. XBP1s ablation mice displayed a similar myocardial lipid disturbance and cardiac phenotypes, which were reversed by EDEM2 overexpression. Mechanistically, the findings obtained from rat cardiomyocytes and human induced pluripotent stem cell–derived cardiomyocytes demonstrated that, in the presence of EDEM2, SEC23A mediated intracellular translocation of ATGL (adipose triglyceride lipase) under fatty acid stimulation, inhibiting ATGL degradation and excessive intracellular lipid droplets. Furthermore, the functional studies supported that EDEM2 prevention of lipid overload occurred in an ATGL-dependent manner. Therapeutically, cardiac XBP1s or EDEM2 restoration mitigated lipid deposition and preserved lipid profiles in the myocardium, thus preventing the development of HFpEF.

**CONCLUSIONS:**

We demonstrate a cardioprotective mechanism regulating myocardial lipid homeostasis. The findings provide a promising therapeutic target to prevent and treat HFpEF, a condition with limited treatment options.

**Clinical Perspective**

**What Is New?**

- This study has uncovered a novel transcriptional target of XBP1s (spliced X-box binding protein 1), EDEM2 (endoplasmic reticulum degradation–enhancing alpha-mannosidase–like protein 2), in cardiomyocytes and its crucial function as an important modulator governing cardiac lipid homeostasis, evidenced by loss- and gain-of-function models.
- This study has provided the molecular and functional evidence that deficiency of XBP1s-EDEM2 is a causative factor of cardiac lipotoxicity, accelerating the onset and development of heart failure in metabolic disorders.
- This study has deciphered that cardiac EDEM2 exerts its cardioprotective role by preserving proper translocation of ATGL (adipose triglyceride lipase) in cells and its physiological action of lipid turnover, preventing and treating heart failure with preserved ejection fraction under prolonged metabolic stress.

**What Are the Clinical Implications?**

- Data gathered from preclinical heart failure with preserved ejection fraction models have supported the causative link between cardiac lipotoxicity and progression of heart failure with preserved ejection fraction.
- Our findings of the molecular link between EDEM2 disruption and intracellular lipid disturbances have provided the proof-of-concept evidence that the XBP1s and EDEM2 pathway is a promising target for mitigating cardiac lipotoxicity.
- Evidence from our functional studies on mice endorses the hypothesis that inhibition of cardiac lipotoxicity can tackle metabolic insults in the heart, which can be a potential strategy in clinics for the prevention and treatment of heart failure in metabolic syndrome.

Metabolic disorders, such as hyperglycemia, hypertension, and dyslipidemia, increase the risk of developing heart failure (HF). HF with preserved ejection fraction (HFpEF) is predominant under metabolic stress, and, in later stages, it can progress to HF with reduced ejection fraction.^1^ Clinical studies have failed to identify effective treatments for HFpEF because of a limited understanding of the underlying mechanisms.

The etiology of HF under metabolic stress is multifactorial and includes lipotoxicity within the myocardium.^2,3^ Lipid droplets (LDs) in cardiomyocytes act as neutral lipid reservoirs providing a fuel source for energy production; however, the myocardium has a limited capability for lipid storage. Cardiac lipotoxicity is characterized by accumulation of lipids, including diglycerides (DGs) and triglycerides (TGs), mitochondrial dysfunction, and oxidative stress, culminating in cardiac energy deprivation.^4–6^ However, the molecular bases driving myocardial lipid overload are poorly understood.

LDs consist of a phospholipid monolayer decorated with proteins for LD biogenesis and degradation.^7^ ATGL (adipose triglyceride lipase) binds to LD surfaces, hydrolyzing TGs to release fatty acids (FAs).^8^ ATGL mutations are involved in diseases characterized by abnormal lipid storage.^9–11^ In the heart, loss of function of ATGL induces cardiac steatosis and mitochondrial dysfunction, contributing to HF.^6,12^ Conversely, reinforced ATGL prevents cardiac lipotoxicity, cardiomyopathy, and cardiac dysfunction.^13,14^ It has been reported that ATGL primarily resides in the endoplasmic reticulum (ER) and is transported to the LD surface through ER-involved vesicle transport^15–17^; however, the underpinning mechanisms in cardiomyocytes are unclear.

EDEMs (endoplasmic reticulum degradation–enhancing alpha-mannosidase–like proteins) play roles in protein modification and degradation and vesicular transportation of proteins.^18^ Among EDEMs, EDEM2 is the initiator of protein quality control and modulator of protein trafficking.^19^ In *Drosophila*, EDEM2 protects against age-related behavioral decline,^20^ whereas in *Caenorhabditis* *elegans*, EDEM2 has been shown to be involved in regulating organism survival and preconditioning against ER stress.^21^ Recent research has demonstrated a link between an EDEM2 variant and monogenic childhood-onset diabetes, likely by blocking insulin production and secretion,^22^ but its role in the heart remains unexplored. EDEM2 is positively correlated with XBP1s (spliced X-box binding protein 1) in HEK293 cells^23^ as a transcription factor modulating genes participating in a wide range of biological processes, whose reduction is detected in metabolic disorder–associated HF.^24,25^

Here, our molecular and functional studies first uncover XBP1s transcriptional regulation of EDEM2 in cardiomyocytes. Mice with loss of XBP1s or EDEM2 were more vulnerable to metabolic stress–induced myocardial lipotoxicity and cardiac dysfunction. Conversely, cardiac XBP1s or EDEM2 restoration alleviated lipid overload in the myocardium and rescued HFpEF. Mechanistic studies revealed that EDEM2 facilitated proper ATGL translocation, inhibiting ATGL degradation upon FA stimulation. Taken together, this study provides functional and phenotypic evidence demonstrating the pivotal function of EDEM2 in the maintenance of cardiac lipid homeostasis. Additionally, it highlights potential therapeutic strategies for the prevention and treatment of HFpEF by targeting cardiac lipotoxicity.

## METHODS

The data, experimental materials, and methods are available from the corresponding author upon reasonable request for purposes of reproducing the results or replicating the procedure. Detailed methods are available in the Supplemental Material.

### Mouse Studies

All animal studies were performed in accordance with the United Kingdom Animals (Scientific Procedures) Act 1986 and were approved by the University of Manchester Ethics Committee. Adeno-associated virus 9 (AAV9) gene delivery was used to overexpress or knock down EDEM2 or XBP1s in male C57BL/6 mice ≈7 weeks of age. Metabolic stress was induced by feeding mice a high-fat diet (HFD), 60% calories from fat, and 0.5 g/L Nω-nitro-L-arginine methyl ester (L-NAME)^25^ for various durations according to the experimental design.

### Human Samples

Fresh human heart slices were provided by a consenting and deidentified donor (United States transplantation network, Novabiosis). Cultured heart slices were stimulated by FAs, approved by the institutional review boards of the University of Louisville.^26^ Additionally, snap-frozen human heart tissues, either purchased from Asterand (BioIVT, UK) or obtained from the United Network for Organ Sharing through the International Institute for the Advancement of Medicine and Novabiosis, were used to perform RNA sequencing and liquid chromatography/mass-spectrometry analyses accordingly.

### Transmission Electron Microscopy

Fresh tissue was fixed in 2.5% glutaraldehyde + 4% formaldehyde in 0.1 M Hepes buffer (pH 7.2). Following post-fixation, dehydration, and epoxy resin embedding, ultrathin sections were cut with a Reichert Ultracut ultramicrotome. Images were taken and analyzed using a Talos L120C transmission electron microscope at 120-kV accelerating voltage with a Ceta CMOS camera.

### Oil Red O Staining

Heart sections or cells were fixed in 10% formalin followed by incubation with oil red O (0.15% in isopropyl alcohol). Sections were rinsed prior to mounting with Immuno HistoMount mounting solution.

### Statistical Analyses

Data are presented as bar/dot plots showing mean±SEM. Where sample sizes were ≥5, the Shapiro-Wilk test was conducted to determine whether data were normally distributed. Normally distributed data were analyzed using ordinary 1-way or 2-way ANOVA followed by appropriate post hoc tests, whereas comparisons between 2 groups were performed using Student *t* test. The non-parametric equivalents were used for skewed data and data sets where sample size was <5. Statistical analysis was performed using the GraphPad Prism 10 software, and *P*<0.05 was considered statistically significant.

## RESULTS

### EDEM2 Is Decreased in Metabolic Disorder–Related Failing Hearts

**Figure 1.**
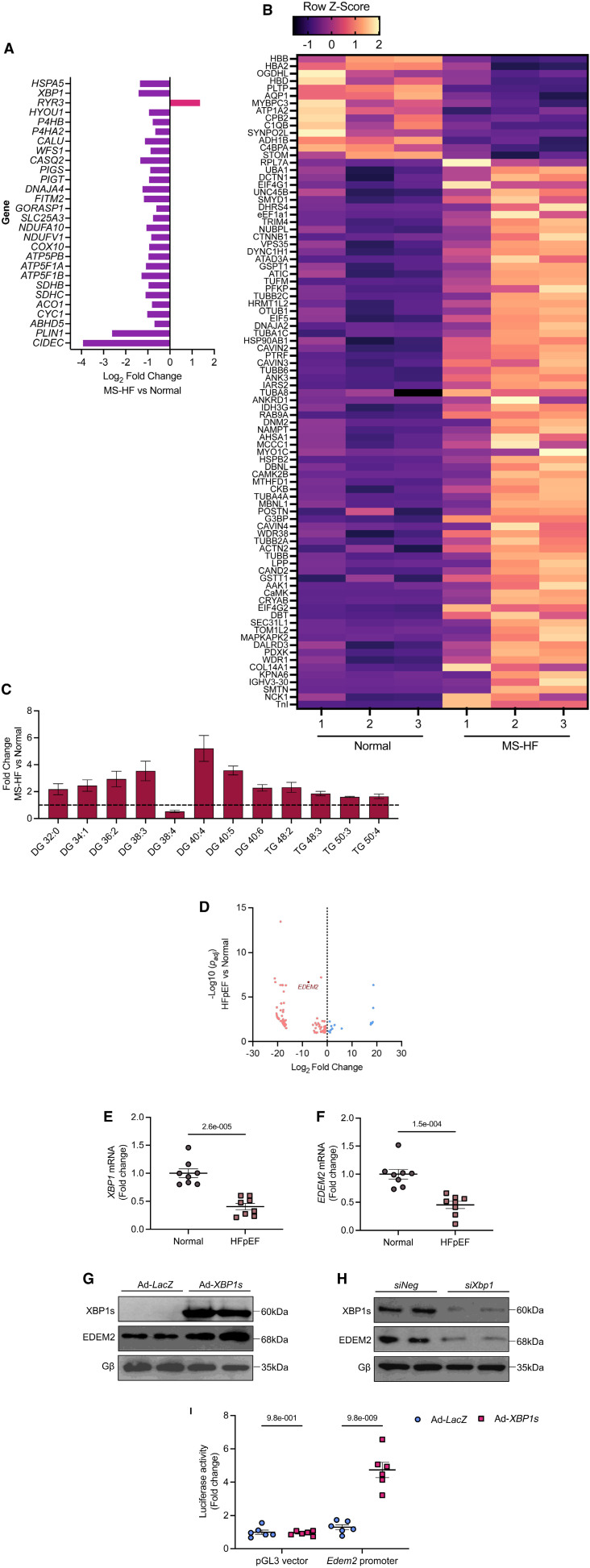
**Endoplasmic reticulum degradation–enhancing alpha-mannosidase–like protein 2 is regulated by spliced X-box binding protein 1 and reduced in hearts with heart failure with preserved ejection fraction. A**, Among the genes with differential expression (*P*_adj_<0.1) identified in human hearts from people with metabolic syndrome (MS) who died of heart failure (HF) compared with normal controls without MS and HF (n=3–5 hearts), genes involved in the endoplasmic reticulum and lipid metabolic function are presented. **B**, Liquid chromatography-mass spectrometry heatmap analysis exhibiting altered proteins in human MS-HF hearts compared with normal controls (n=3 hearts; *P*_*adj*_<0.05). **C**, Lipidomics displaying significantly changed diglycerides and triglycerides in the human heart under metabolic stress (n=3–5 hearts). **D**, RNA sequencing demonstrates the genes with differential expression in human hearts with heart failure with preserved ejection fraction (n=4–6 hearts; *P*_*adj*_<0.1). **E** and **F**, Quantitative PCR of *XBP1* (**E**) and quantitative PCR of *EDEM2* in human hearts with heart failure with preserved ejection fraction (**F**; n=8). **G** and **H**, Immunoblots demonstrating that XBP1s (spliced X-box binding protein 1) overexpression achieved by an adenovirus expressing XBP1s increased EDEM2 (endoplasmic reticulum degradation–enhancing alpha-mannosidase–like protein 2) levels (Ad-*LacZ*: control; **G**) and XBP1s reduction (*siXbp1*) decreased EDEM2 levels in neonatal rat cardiomyocytes (**H**). Gβ is the loading control. **I**, *Edem2* promotor luciferase activity was augmented by XBP1s overexpression (n=6 experiments). Data are presented as mean±SEM. *P* values were calculated using a Mann-Whitney test (**C**), an unpaired Student t test (**E** and **F**), or a 2-way ANOVA with Šidák post hoc tests (**I**). *siNeg* indicates siNegative.

To assess the alterations of cardiac genes in metabolic disorders, we performed RNA sequencing on human hearts from people diagnosed with metabolic syndrome along with various cardiovascular complications, such as ischemic cardiomyopathy, arrhythmic cardiac arrest, coronary artery disease, or congestive HF. Four hundred eighty-eight genes were considered as genes with differential expression (*P*_*adj*_<0.1, 3 normal and 5 metabolic syndrome-associated HF) in metabolic stress–associated failing hearts (Table S1), and pathway enrichment analyses highlighted changes in prominent clusters, including lipid metabolism regulation (Kyoto Encyclopedia of Genes and Genomes and Reactome) and ER components and function (gene ontology cellular component and cellular biological process; Figure S1A through S1D). Specifically, the genes involved in maintaining ER function, including *XBP1*, as well as those responsible for lipid metabolism are shown in Figure 1A. Furthermore, proteomics of human heart lysates followed by pathway analyses of enriched proteins also revealed alterations in ER-related factors and lipid metabolism in the failing heart (Figure S1E through S1H). Particularly, dysregulated proteins (*P*_*adj*_<0.05) included factors working for lipid homeostasis and ER function (Figure 1B). Moreover, lipidomics detected more DGs and TGs in these hearts (Figure 1C), accompanied by increased oxidative stress (Figure S1I). These data infer a relevance between ER dysregulation and lipid abnormalities in metabolic disorder–associated human hearts.

HFpEF accounts for more than half of HF cases in metabolic disorders^1^; we therefore assessed gene and lipid profiles in HFpEF hearts. Proteomics of mouse heart lysates detected downregulation of proteins (*P*_*adj*_<0.05) participating in ER function (Figure S2A), whereas lipidomics detected significantly increased lipids, including DGs and TGs, in mouse HFpEF hearts induced by the 2-hit method (HFD and L-NAME; Figure S2B).^25^ Strikingly, RNA sequencing of human hearts (4 normal and 6 HFpEF) detected that *EDEM2* was significantly reduced in HFpEF (Figure 1D). Convincingly, by assessing potential targets of XBP1s, the simultaneous reduction in cardiac *XBP1* and *EDEM2* in human HFpEF hearts was validated using quantitative polymerase chain reaction (Figure 1E and 1F; Figure S3). Next, XBP1s positive regulation of EDEM2 was determined in neonatal rat cardiomyocytes (Figure 1G and 1H; Figure S4A). Because in silico analyses identified that XBP1s has putative binding sites in the *Edem2* promoter region across different mammalian species (Figure S4B), the fact that XBP1s transcriptionally regulates *Edem2* was further evidenced by luciferase reporter assays (Figure 1I) and chromatin immunoprecipitation assays (Figure S4C).

Importantly, downregulation of EDEM2 was observed in hearts from various metabolically stressed human and mouse models (Figure S5A through S5E). Likewise, when subjected to high-FA treatment, cultured human heart slices also exhibited a notable decline in EDEM2 (Figure S5F). Analysis using the Cardiovascular Disease Knowledge Portal provided genetic evidence supporting the involvement of EDEM2 in cardiovascular diseases and lipid regulation (Figure S5G). Thus, we decided to investigate the role of EDEM2 in myocardial lipid homeostasis and cardiac function under metabolic stress.

### EDEM2 Deficiency Exacerbates Cardiac Lipotoxicity

**Figure 2.**
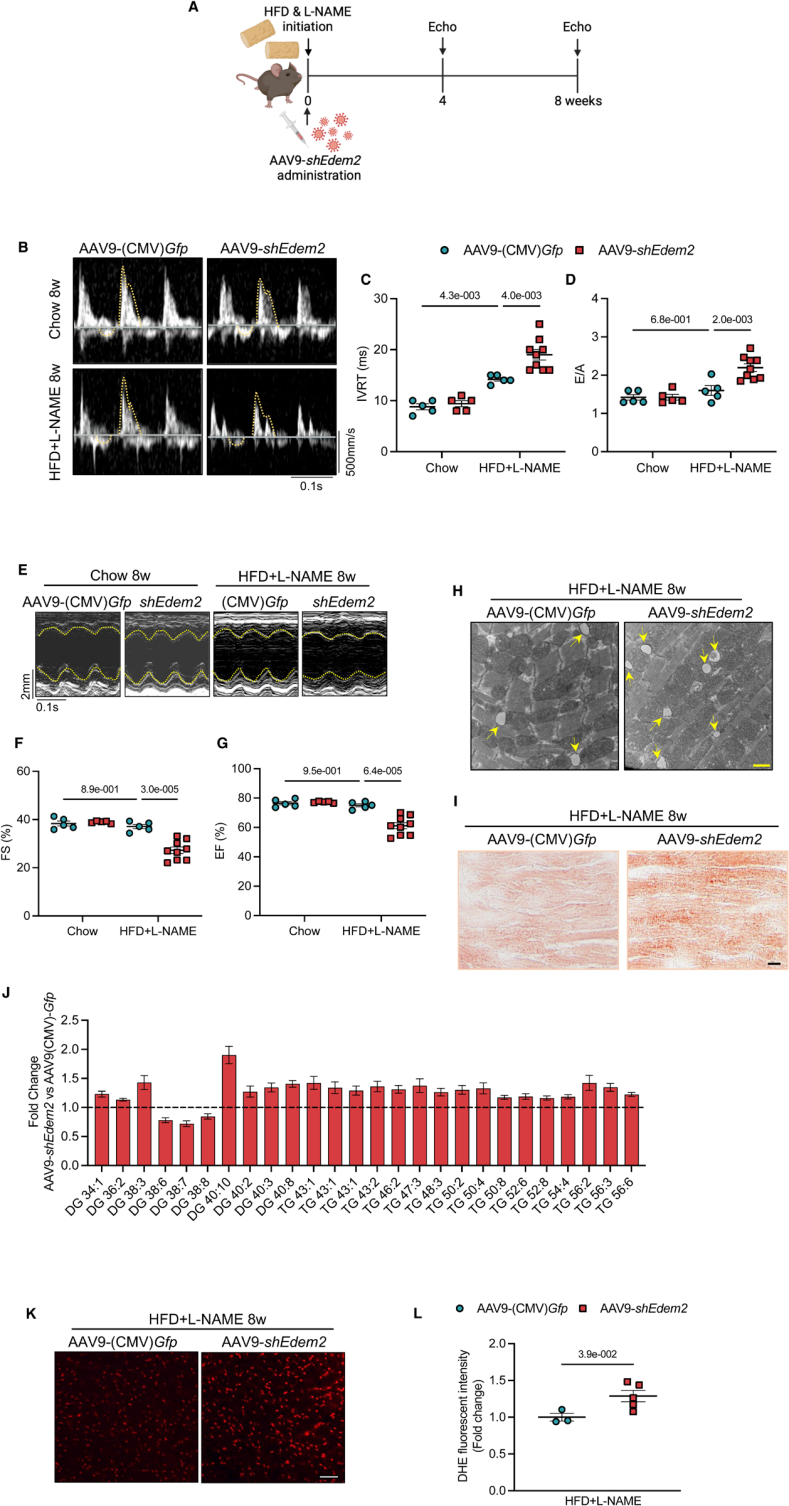
**Endoplasmic reticulum degradation–enhancing alpha-mannosidase–like protein 2 deficiency induces lipotoxicity and impairs cardiac function under metabolic stress. A**, Schematic of the experimental design. **B**, Representative pulsed-wave Doppler tracings. **C**, Isovolumic relaxation time (n=5–9 mice). **D**, Ratio of peak velocity blood flow from left ventricular relaxation in early diastole to that in late diastole (n=5–9 mice). **E**, Representative left ventricular M-mode echocardiographic tracings in short-axis view. **F**, Percentage of left ventricular fractional shortening (FS%; n=5-9 mice). **G**, Percentage of ejection fraction (n=5–9 mice). **H**, Representative transmission electron microscopy images; arrows indicate lipid droplets (scale bar=1 µm). **I**, Representative oil red O staining (scale bar=20 µm). **J**, Lipidomic analysis displaying diglycerides and triglycerides significantly altered in EDEM2 (endoplasmic reticulum degradation–enhancing alpha-mannosidase–like protein 2) knockdown hearts compared with control mice exposed to metabolic stress (n=5–9 hearts; *P*<0.05). **K**, Representative images of dihydroethidium staining (scale bar=50 µm). **L**, Quantification of dihydroethidium intensity (n=3–5 hearts). Data are presented as mean±SEM. *P* values were calculated using a 2-way ANOVA with Tukey post hoc tests (**C**, **D**, **F**, and **G**), an unpaired Student *t* test (**J**), or a Mann-Whitney test (**L**). DG indicates diglyceride; DHE, dihydroethidium; E/A, ratio of peak velocity blood flow from left ventricular relaxation in early diastole to that in late diastole; FS%, percentage of left ventricular fractional shortening; IVRT, isovolumic relaxation time; and TG, triglyceride.

To evaluate the link between EDEM2 reduction and cardiac lipotoxicity, EDEM2 was knocked down in mice using AAV9 delivery of *shEdem2* (Figure S6A and S6B). Following an HFpEF-like condition for 8 weeks (Figure 2A), similar systemic metabolic profiles were detected in both controls and EDEM2 knockdown mice (Figure S6C through S6E); however, diastolic dysfunction, a key element for the diagnosis of HFpEF,^1^ occurred earlier and more severely in mice lacking EDEM2, as evidenced by increased isovolumic relaxation time (*P*=4.0e−003 compared with control mice under metabolic stress) and the ratio of peak velocity blood flow from left ventricular relaxation in early diastole to that in late diastole (*P*=2.0e−003), and pulmonary edema (Table S2; Figure 2B through 2D). Additionally, EDEM2 deficiency led to minor systolic dysfunction under stress, evidenced by decreased fractional shortening (*P*=3.0e−005) and ejection fraction (*P*=6.4e−005) (Table S2; Figure 2E through 2G). Of note, a similar phenomenon was reproduced in the mice subjected to stress induced by HFD alone (Figure S7), suggesting that EDEM2 deficiency makes the heart more susceptible to cardiac dysfunction upon metabolic stress, primarily responding to a fatty diet.

EDEM2 loss triggered pathological cardiac remodeling, indicated by hypertrophic cardiomyocytes (Figure S8A), higher levels of a pathological hypertrophy marker (*Nppb*), and fibrotic genes (*Col1a2* and *Col3a1*; Figure S8B). Noticeably, transmission electron microscopy detected more LDs in the EDEM2 knockdown myocardium (Figure 2H), in line with elevated neutral lipids determined by oil red O staining (Figure 2I). Lipidomics of the myocardium revealed higher levels of long-chain DGs and TGs correlating with the loss of EDEM2 (Figure 2J). As a result, reactive oxygen species were higher in hearts with EDEM2 loss (Figure 2K and 2L). These results demonstrate that EDEM2 deficiency–triggered cardiac lipotoxicity contributes to HFpEF. Additionally, EDEM2 knockdown led to decreases in XBP1s and ATF6 (activating transcription factor 6; Figure S8C), implying that EDEM2 reduction deteriorates ER dysfunction, likely via a vicious cycle.

### EDEM2 Loss Leads to Lipid Mishandling via ATGL Retention in the ER

**Figure 3.**
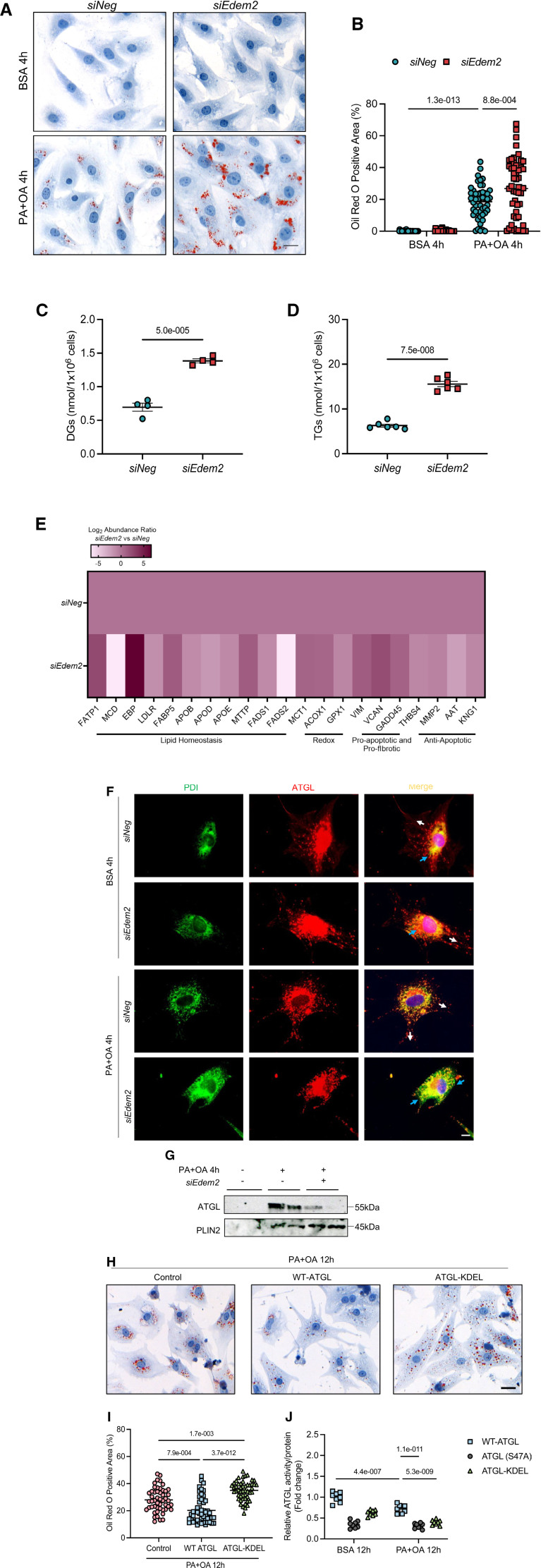
**Endoplasmic reticulum degradation–enhancing alpha-mannosidase–like protein 2 knockdown increases lipid droplet accumulation and endoplasmic reticulum retention of adipose triglyceride lipase in cardiomyocytes with fatty acid stimulation. A**, Representative images of oil red O staining of neonatal rat cardiomyocytes (NRCMs) with EDEM2 (endoplasmic reticulum degradation–enhancing alpha-mannosidase–like protein 2) knockdown (*siEdem2*) with 4 hours of palmitic acid and oleic acid stress (300 μM; scale bar=20 µm). **B**, Percentage of oil red O–positive area in NRCMs (n=50 cells collected from 3 separate experiments). **C** and **D**, Diglyceride (**C**) and triglyceride (**D**) contents in H9C2 myoblasts subjected to stress of palmitic acid and oleic acid for 4 hours (n=4–6 experiments). **E**, Liquid chromatography-mass spectrometry analysis showing proteins significantly changed in EDEM2 knockdown H9C2s (*P*_*adj*_<0.05) under stress for 4 hours (n=3 experiments). **F**, ATGL (adipose triglyceride lipase) distribution in NRCMs. Endoplasmic reticulum retention (blue arrows) is indicated by an endoplasmic reticulum marker, PDI (protein disulfide isomerase [green]); white arrows indicate cytosol-diffused ATGL (red). Nuclei were stained with 4',6-diamidino-2-phenylindole (DAPI; blue; scale bar=20 µm). **G**, Levels of ATGL on isolated lipid droplets (perilipin 2 as a lipid droplet indicator) from 5×10^7^ H9C2 cells. **H**, Representative images of oil red O staining of NRCMs overexpressing wild-type or endoplasmic reticulum–retained ATGL (scale bar=20 µm). **I**, Quantification of oil red O–positive area in NRCMs (n=50 cells collected from 3 separate experiments). **J**, ATGL lipase activity assay in whole-cell extracts from H9C2 cells expressing wild-type ATGL, mutant ATGL (S47A), or endoplasmic reticulum–retained ATGL in the absence and presence of fatty acid stress (n=8 experiments). Data are presented as mean±SEM. *P* values were calculated using a 2-way ANOVA with Tukey post hoc tests (**B**) or Šidák post hoc tests (**J**), a Mann-Whitney test (**C**), an unpaired Student *t* test (**D**), or a Kruskal-Wallis test with Dunn’s post hoc tests (**I**). ATGL indicates adipose triglyceride lipase; ATGL-KDEL, endoplasmic reticulum–retained ATGL; DG, diglyceride; OA, oleic acid; PA, palmitic acid; *siNeg*, siNegative; TG, triglyceride; and WT, wild type.

According to the similar alterations of cardiac performance and LD accumulation in EDEM2-deficient mice under 1-hit (HFD only) and 2-hit (HFD+L-NAME) stressed models, excess FAs are likely the primary cause impairing lipid metabolism.^27^ Thus, we used FAs to stimulate cells, investigating the underlying mechanisms. Concomitant decreases in XBP1s and EDEM2 were detected in neonatal rat cardiomyocytes upon long-term FA stress (Figure S9). Therefore, we used EDEM2 knockdown cells under short-term FAs to investigate the correlation between EDEM2 reduction and lipid overload. Consistent with observations in the myocardium, cells with EDEM2 ablation (Figure S10A) displayed LD accumulation upon acute FA stimulation (Figure 3A and 3B; Figure S10B and S10C), accompanied by elevated DGs and TGs (Figure 3C and 3D). When assessing the intracellular pathways affected by EDEM2 loss, proteomics detected lipid metabolism– and ER-related pathways largely influenced by EDEM2 deficiency (*P*_*adj*_<0.05) (Figure S10D through S10G). Specifically, proteins mediating lipid clearance were dysregulated on account of EDEM2 disruption. EDEM2 knockdown cells also exhibited an increase in oxidative stress indicators and pro-fibrotic and apoptotic factors and a decrease in antifibrotic and antiapoptotic factors (Figure 3E). These data support that EDEM2 deficiency alters lipid profiles in cardiomyocytes under metabolic-like stress.

Under normal physiological conditions, elevated intracellular LDs are attributable to enhanced lipid uptake and synthesis, decreased lipolysis and usage, or both. Thus, we next unraveled the underlying mechanisms of LD accumulation caused by loss of EDEM2. We first assessed the key genes participating in lipid metabolism. At the transcript level, the genes involved in FA uptake, breakdown, and oxidation were dysregulated (Figure S11A), but their protein levels were comparable, although CPT1B (carnitine palmitoyltransferase 1B) was reduced in EDEM2 knockdown hearts (Figure S11B). Of note, *Pnpla2* (the ATGL transcript) was similar in the presence or absence of EDEM2 (Figure S11A); however, its cytosolic expression was reduced, whereas the ER portion of ATGL was increased (Figure S11C) with EDEM2 reduction. In times of energy demand, TGs are hydrolyzed to liberate FAs by LD surface lipases. ATGL is the rate-limiting and most influential lipase; it is first embedded in the ER membrane through a hydrophobic domain and then shuttles to the LDs.^15^ Thus, we next examined the impact of EDEM2 on ATGL intracellular localization.

Notably, ATGL was diffused in the cytosol in both neonatal rat cardiomyocytes and H9C2 myoblasts upon short-term FA stress, whereas it was more retained in the ER in EDEM2-deficient cells (Figure 3F; Figure S11D and S11E). Additionally, isolated LDs of the EDEM2 knockdown cells showed lessened distribution of ATGL on the LDs (Figure 3G). Next, we determined whether improperly localized ATGL is easy to degrade. The proteasome inhibitor MG132 blocked ATGL reduction (Figure S11F), so increased ubiquitinated ATGL attributable to EDEM2 loss (Figure S11G) affirmed more degradation of ATGL under an EDEM2-deficit condition. A similar phenomenon was observed in hearts lacking EDEM2 (Figure S11H). These observations indicate that EDEM2 depletion restricts physiological release of ATGL from the ER, ultimately accelerating its degradation.

Prompted by the reduced ATGL activity in myocardium with EDEM2 deficiency (Figure S12), we assessed whether it is the ER retention of ATGL that attenuates lipolysis. We used the ATGL-Lys-Asp-Glu-Leu (KDEL) plasmid to constrain ATGL in the ER (Figure S13A and 13B), which augmented ATGL ubiquitination (Figure S13C). Upon prolonged FA stress, compared with wild-type ATGL, ATGL-KDEL substantially exaggerated LD deposition (Figure 3H and 3I), likely attributable to lower ATGL activity (Figure 3J). A similar result was led by enzymatically dead ATGL (ATGL-S47A; Figure 3J). Finally, ATGL overexpression rescued the impairment of ATP concentration in EDEM2 knockdown cells, whereas the mutant ATGL forms (ATGL-S47A or ATGL-N172K as functionally dead forms) did not,^13^ consistent with the observation in cells treated with an CPT1 inhibitor (etomoxir; Figure S14). Collectively, these data signify that EDEM2 loss constrains endogenous ATGL translocation and accelerates ATGL degradation, exacerbating lipid excess.

### EDEM2 Loss–Induced Cardiac Lipotoxicity Is Attributable to ATGL Dysregulation

We gained further functional evidence that EDEM2 deficiency–induced lipid overload arises because of ATGL dysregulation. EDEM2 knockdown mice were subjected to HFpEF-like stress for 4 weeks, followed by administration of various drugs (Figure S15A). Alda-1 is an agonist for aldehyde dehydrogenase 2, which diminishes lipid overload.^28^ Four-week treatment of Alda-1 reversed cardiac dysfunction in EDEM2 knockdown mice (Figure S15B through S15E; Table S3). In addition, as a regulator of lipid homeostasis,^29^ oxytocin treatment also improved cardiac function despite EDEM2 abrogation (Figure S15B through S15E; Table S3). Both drugs attenuated cardiac hypertrophy (Figure S15F), LD deposition, and oxidative stress (Figure S15G and S15H). In contrast, SR-4995 did not show beneficial effects on cardiac function or cardiac lipotoxicity (Figure S15B through S15H; Table S3). ATGL regulates lipolysis by activation of ABHD5 (alpha/beta-hydrolase domain–containing 5, also known as CGI-58). SR-4995 is a ligand of ABHD5 that enhances its interaction with ATGL on the LD surface.^30^ Notably, SR-4995 failed to counteract myocardial lipotoxicity in either control mice under HFpEF-like stress, where EDEM2 was reduced (Figure S5D), or EDEM2 knockouts. These data support the fact that ATGL is dysregulated because of EDEM2 loss, whereas the cardioprotective effects of blocking lipotoxicity by Alda-1 and oxytocin occur via mechanisms bypassing EDEM2.

### EDEM2 Prevents Lipid Overload in Cardiomyocytes Under FA Stress

**Figure 4.**
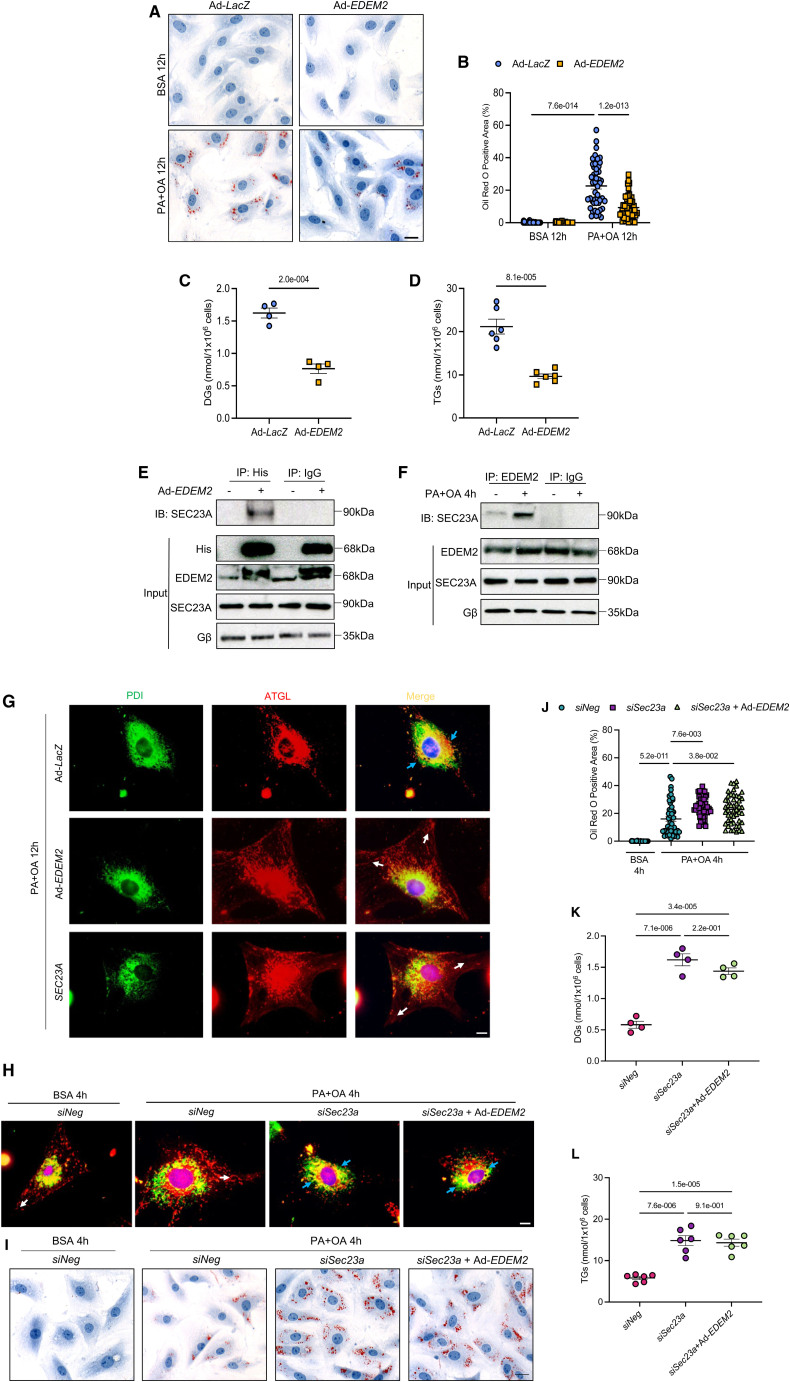
**Endoplasmic reticulum degradation–enhancing alpha-mannosidase–like protein 2 overexpression decreases lipid droplet accumulation and triggers SEC23A-mediated adipose triglyceride lipase release from the endoplasmic reticulum in cardiomyocytes. A**, Representative images of oil red O staining of neonatal rat cardiomyocytes with EDEM2 (endoplasmic reticulum degradation–enhancing alpha-mannosidase–like protein 2) overexpression with stimulation of palmitic acid and oleic acid stress (300 μM) for 12 hours (scale bar=20 µm). **B**, Percentage of oil red O–positive areas in cells (n=50 cells collected from 3 separate experiments). **C** and **D**, Diglycerides (**C**) and triglycerides (**D**) in H9C2 cells (n=4–6 experiments) under stress for 12 hours. **E**, Co-immunoprecipitation demonstrating the association of EDEM2 and SEC23A in EDEM2-overexpressing H9C2 cells. **F**, Co-immunoprecipitation displaying endogenous EDEM2 and SEC23A association in H9C2 upon acute fatty acid stimulation. **G**, ATGL (adipose triglyceride lipase; red) release from the endoplasmic reticulum (ER; PDI [protein disulfide isomerase], an ER marker, is shown in green) as shown by white arrows in EDEM2- or SEC23A-overexpressing neonatal rat cardiomyocytes (blue arrows indicate ER-retained ATGL), nuclei were stained with 4',6-diamidino-2-phenylindole (DAPI; blue; scale bar=20 µm). **H**, ATGL (red) localization in neonatal rat cardiomyocytes with SEC23A deficiency (PDI, an ER marker, is shown in green). White arrows indicate cytosolic trafficking of ATGL, whereas blue arrows indicate ER-retained ATGL (scale bar=20 µm). **I**, Representative images of oil red O staining of neonatal rat cardiomyocytes with SEC23A knockdown (*siSec23a*) in the absence and presence of EDEM2 overexpression with 4 hours of stimulation (palmitic acid and oleic acid, 300 μM; scale bar=20 µm). **J**, Percentage of oil red O–positive areas in cells (n= 50 cells collected from 3 separate experiments). **K** and **L**, diglycerides (**K**) and triglycerides (**L**) in H9C2 cells under stress for 4 hours (n=4–6 experiments). Data are presented as mean±SEM. *P* values were calculated using a 2-way ANOVA with Tukey post hoc tests (**B**), a Mann-Whitney test (**C**), an unpaired Student *t* test (**D**), a 1-way ANOVA with Tukey post hoc tests (**J**), or a Kruskal-Wallis test with Dunn’s post hoc tests (**K** and **L**). DG indicates diglyceride; IP, immunoprecipitation; OA, oleic acid; PA, palmitic acid; *siNeg*, siNegative; and TG, triglyceride.

Led by these observations, we assessed whether EDEM2 can inhibit lipid overload under long-term stimulation with FAs. First, EDEM2 overexpression, achieved by an adenovirus expressing *EDEM2* (Figure S16A and S16B), mitigated LD deposition in cardiomyocytes and H9C2 cells following 12 hours of FA treatment (*P*=1.2e−013; Figure 4A and 4B; Figure S16C and S16D). Lower levels of DGs (*P*=2.0e−004) and TGs (*P*=8.1e−005) in EDEM2-overexpressing cells supported its role in preventing lipid overload (Figure 4C and 4D). Of note, long-term stimulation resulted in ATGL retainment in the ER, whereas EDEM2 overexpression promoted its release from the ER (Figure S16E and S16F). More importantly, the reduction in ATP following long-term FA treatment was rescued by EDEM2 overexpression, akin to the effects of XBP1s augmentation (either by Ad-*XBP1s* or a pharmacological compound, IXA4^31^). However, ATGL knockdown blocked the positive effects on ATP in EDEM2-overexpressing cells (Figure S16G), supporting that EDEM2 preservation of lipid equilibrium is mediated via ATGL.

### EDEM2 Is Required for SEC23A-Mediated ATGL Translocation

Because colocalization of EDEM2 and CANX (calnexin, an ER marker) or ERGIC53 (ER-Golgi intermediate compartment 53, an ER-Golgi intermediate compartment marker) was observed (Figure S17A), we investigated how EDEM2 mediates ATGL release from the ER. Pull-down proteomics of EDEM2-overexpressing cell lysates (Figure S17B) identified 26 proteins that interact with EDEM2 (Figure S17C). Among them was SEC23A, a component of COPII (coat protein complex II), which is involved in facilitating the translocation of enzymes regulating lipid metabolism to the LDs.^15,32^ Short-term FA treatment stimulated an increase in ATGL levels, which was attenuated in the isolated LDs from SEC23A knockdown cells, leading to more LDs (Figure S18A through S18C). Next, we demonstrated the interaction of EDEM2 and SEC23A in EDEM2-overexpressing cardiomyocytes (Figure 4E) and the association of endogenous EDEM2 and SEC23A under acute FA stimulation (Figure 4F), indicative of their synergistic function to combat short-term stress. More importantly, either EDEM2 or SEC23A overexpression ensured ATGL release from the ER during prolonged stress (Figure 4G). Conversely, SEC23A loss retained ATGL in the ER, which failed to be rescued by EDEM2 restoration (Figure 4H). As a consequence, excess lipid accumulation coupled with cytotoxic effects was observed by SEC23A abrogation (Figure 4I through 4L; Figure S18D). Remarkably, SEC23A knockdown lowered intracellular ATP, which was similarly observed by treatment with brefeldin A (an inhibitor of protein trafficking from the ER; Figure S18E). Altogether, the data show that EDEM2 is involved in SEC23A-mediated ATGL translocation.

### Restoration of EDEM2 Has Therapeutic Effects on HFpEF

**Figure 5.**
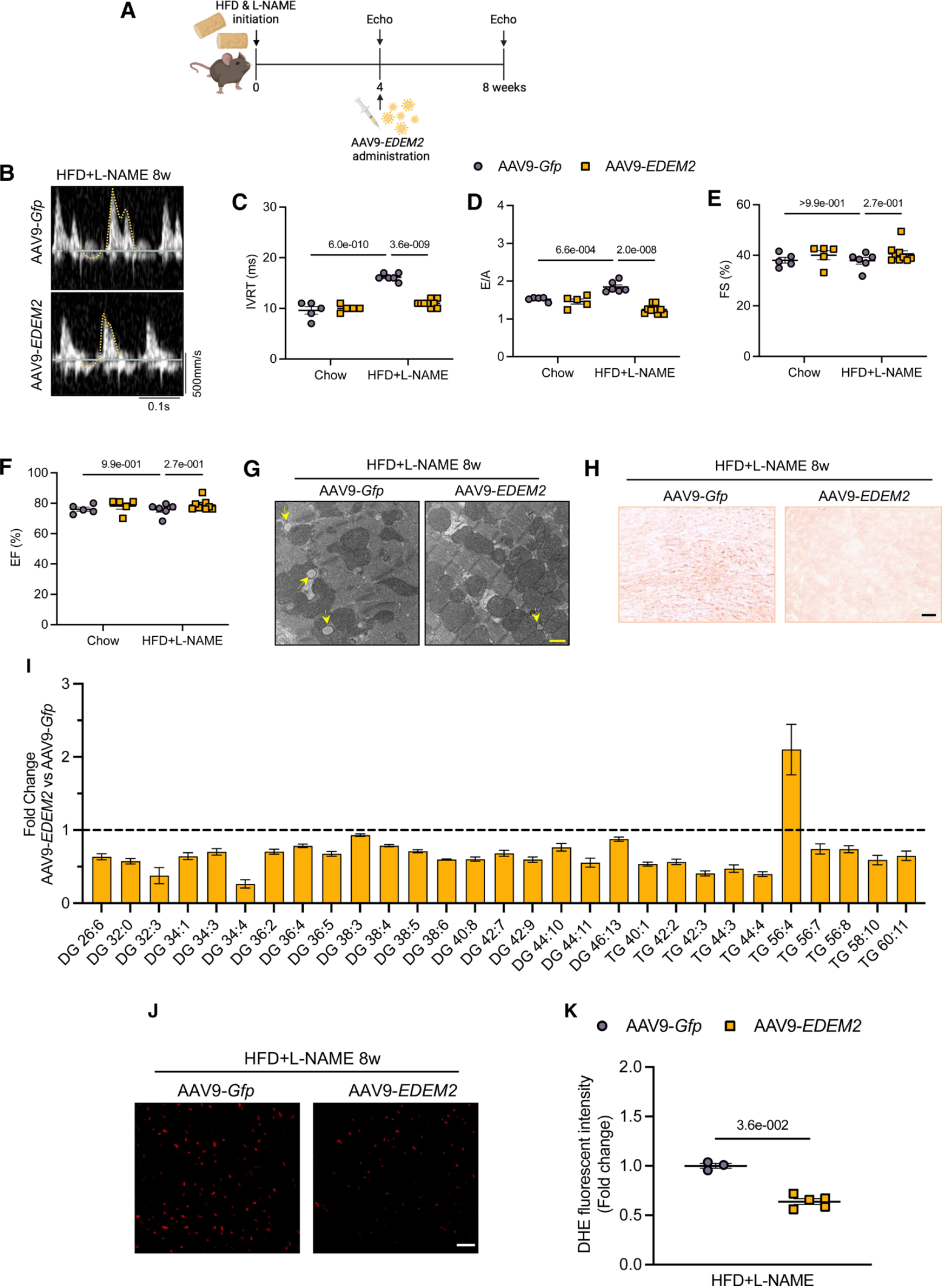
**Endoplasmic reticulum degradation–enhancing alpha-mannosidase–like protein 2 overexpression alleviates cardiac lipotoxicity and heart failure with preserved ejection fraction upon metabolic stress. A**, Schematic of the experimental design. **B**, Representative pulsed-wave Doppler tracings. **C**, Isovolumic relaxation time. **D**, Ratio of peak velocity blood flow in early diastole to late diastole. **E**, Percentage of left ventricular fractional shortening. **F**, Percentage of ejection fraction (n=5–9 mice). **G**, Representative transmission electron microscopy images; arrows indicate lipid droplets (scale bar=1 µm). **H**, Oil red O staining (scale bar=20 µm). **I**, Lipidomics analysis of mice hearts displaying diglycerides and triglycerides significantly altered in EDEM2 (endoplasmic reticulum degradation–enhancing alpha-mannosidase–like protein 2)-overexpressing hearts compared with control mice exposed to metabolic stress induced by a high-fat diet and Nω-nitro-L-arginine methyl ester (n=4–5 hearts, *P*<0.05). **J**, Representative dihydroethidium staining images (scale bar=50 µm). **K**, Quantification of dihydroethidium intensity (n=3–5 hearts). Data are presented as mean±SEM. *P* values were calculated using a 2-way ANOVA with Šidák post hoc tests (**C** to **F**) or a Mann-Whitney test (**I** and **K**). DG indicates diglyceride; DHE, dihydroethidium; E/A, ratio of peak velocity blood flow in early diastole to late diastole; EF%, percentage of ejection fraction; FS%, percentage of left ventricular fractional shortening; HFD, high-fat diet; IVRT, isovolumic relaxation time; L-NAME, Nω-nitro-L-arginine methyl ester; and TG, triglyceride.

Furthermore, we obtained functional evidence regarding the treatment potential of restoring EDEM2 to sustain cardiac lipid homeostasis and cardiac function under metabolic stress. Mice under metabolic stress were injected with AAV9-delivering human troponin T promoter-driven *EDEM2* to reinstate EDEM2 in the myocardium (Figure S19A and S19B; Figure 5A). Although cardiac EDEM2 overexpression did not affect metabolic profiles (Figure S19C and S19D), it mitigated diastolic cardiac dysfunction (*P*=3.6e−009 for isovolumic relaxation time and *P*=2.0e−008 for the ratio of peak velocity blood flow from left ventricular relaxation in early diastole to that in late diastole compared with control mice) under metabolic stress caused by either HFpEF-like stress (Figure 5B through 5F; Table S4) or HFD feeding alone for 8 weeks (Figure S20). Of note, transmission electron microscopy images showed fewer LDs in the EDEM2-overexpressing myocardium (Figure 5G), which was in line with observations of oil red O staining (Figure 5H). Furthermore, lipidomics of the myocardium revealed that reinforced EDEM2 reduced the content of DGs and TGs upon 8 weeks of stress (Figure 5I), concomitant with attenuated reactive oxygen species levels (Figure 5J and 5K). Cardiac pathological remodeling was attenuated by EDEM2 overexpression (Figure S21A and S21B). Despite few impacts on the genes involved in lipid metabolism compared with control mice (Figure S21C), EDEM2 overexpression reduced ER distribution of ATGL and enhanced its cytosolic levels (Figure S21D). Correspondingly, ubiquitinated ATGL was reduced by EDEM2 overexpression (Figure S21D and S21E), confirming that EDEM2-facilitated ATGL release from the ER impedes its degradation.

For a further attempt, we assessed the outcomes of restoring cardiac EDEM2 in response to a longer-term HFpEF-like stress up to 12 weeks (Figure S22A). Regardless of unaffected metabolic profiles (Figure S22B through S22D), impaired cardiac diastolic function (Figure S22E and S22F; Table S4), lipid overload coupled with oxidative stress (Figure S22G through S22J), and cardiac remodeling (Figure S22K and S22L) were significantly prevented by EDEM2 overexpression. Assessments of transcripts (Figure S22M) and protein levels (Figure S22N) confirmed that ATGL protein expression was preserved in EDEM2-overexpressing myocardium. In accordance with this observation, ATGL activity was sustained because of EDEM2 maintenance (Figure S22O). Additionally, the levels of XBP1s, ATF6, and ATF4 were also impacted by EDEM2 overexpression, indicative of sustained ER adaptive response (Figure S22P). Taken together, these results substantiate the notion that cardiac EDEM2 prevents the progression of cardiac dysfunction, at least partially, by alleviating lipotoxicity.

### XBP1s Elicits Beneficial Effects on Cardiac Lipid Homeostasis and Function in an EDEM2-Dependent Manner

**Figure 6.**
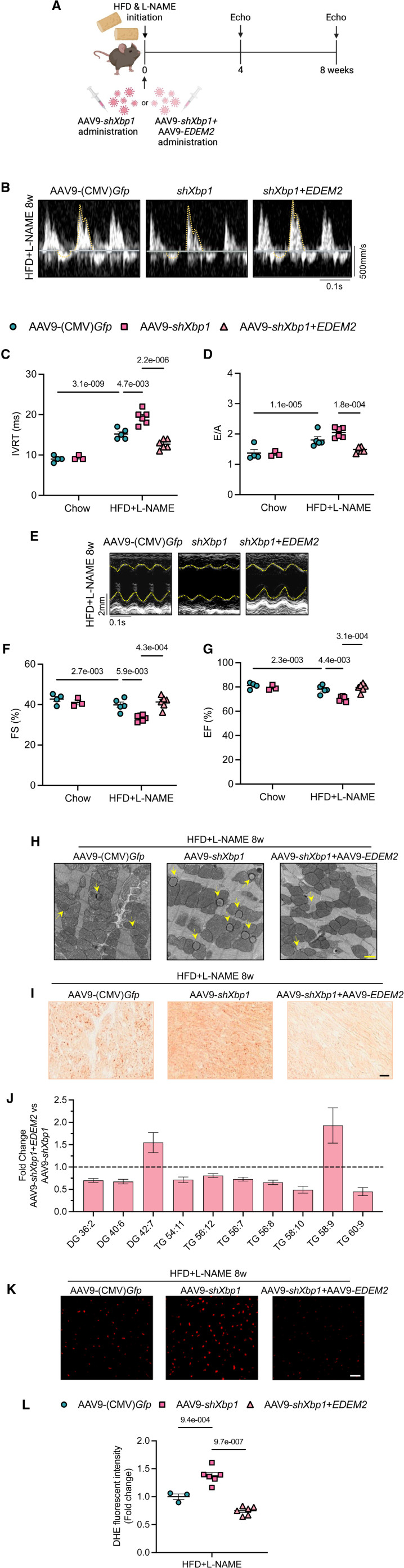
**Spliced X**-b**ox binding protein 1 loss–induced cardiac lipid overload under metabolic stress is rescued by endoplasmic reticulum degradation–enhancing alpha-mannosidase–like protein 2 overexpression. A**, Schematic of the experimental design. **B**, Representative pulsed-wave Doppler tracings. **C**, Isovolumic relaxation time. **D**, Ratio of peak velocity blood flow in early diastole to late diastole obtained from pulsed-wave Doppler. **E**, Representative left ventricular M-mode echocardiographic tracings in short-axis view. **F** and **G**, Percentage of left ventricular fractional shortening (**F**) and percentage of ejection fraction obtained from M-mode echocardiography (n=4–6 mice). **H**, Representative transmission electron microscopy images (arrows indicate lipid droplets; scale bar=1 µm). **I**, Oil red O staining (scale bar=20 µm). **J**, Lipidomics showing significantly changed diglycerides and triglycerides by EDEM2 (endoplasmic reticulum degradation–enhancing alpha-mannosidase–like protein 2) restoration in hearts with loss of XBP1s (spliced X-box binding protein 1) compared with hearts with loss of XBP1s under metabolic stress induced by a high-fat diet and Nω-nitro-L-arginine methyl ester (n=4–5 hearts, *P*<0.05). **K**, Representative images of dihydroethidium staining (scale bar=50 µm). **L**, Quantification of dihydroethidium intensity (n=3–6 hearts). Data are presented as mean±SEM. *P* values were calculated using a 2-way ANOVA with Šidák post hoc tests (**C**, **D**, **F**, **and G**), a Mann-Whitney test (**J**), or a Kruskal-Wallis test with Dunn’s post hoc tests (**L**). DG indicates diglyceride; DHE, dihydroethidium; E/A, ratio of peak velocity blood flow in early diastole to late diastole; EF%, percentage of ejection fraction; FS%, percentage of left ventricular fractional shortening; HFD, high-fat diet; IVRT, isovolumic relaxation time; L-NAME, Nω-nitro-L-arginine methyl ester; and TG, triglyceride.

Given that EDEM2 was identified as a novel transcriptional effector of XBP1s, we sought functional evidence to affirm XBP1s regulation of EDEM2 governing cardiac lipid homeostasis. First, XBP1s was abrogated (Figure S23A and S23B), imitating the condition where XBP1s was reduced in the heart under long-term metabolic stress (Figure 1A). Meanwhile, in the presence or absence of EDEM2 overexpression, the mice were subjected to an HFpEF-like condition for 8 weeks (Figure 6A). With unaffected metabolic profiles (Figure S23C and S23D), XBP1s deficiency prompted cardiac dysfunction, akin to the phenomenon observed in mice with EDEM2 loss. Remarkably, EDEM2 reinforcement rescued the deleterious effects (Figure 6B through 6G; Table S5), particularly prohibiting lipid overload (Figure 6H through 6J), oxidative stress, and cardiac hypertrophy (Figure 6K and 6L; Figure S23E) resulting from XBP1s disruption.

**Figure 7.**
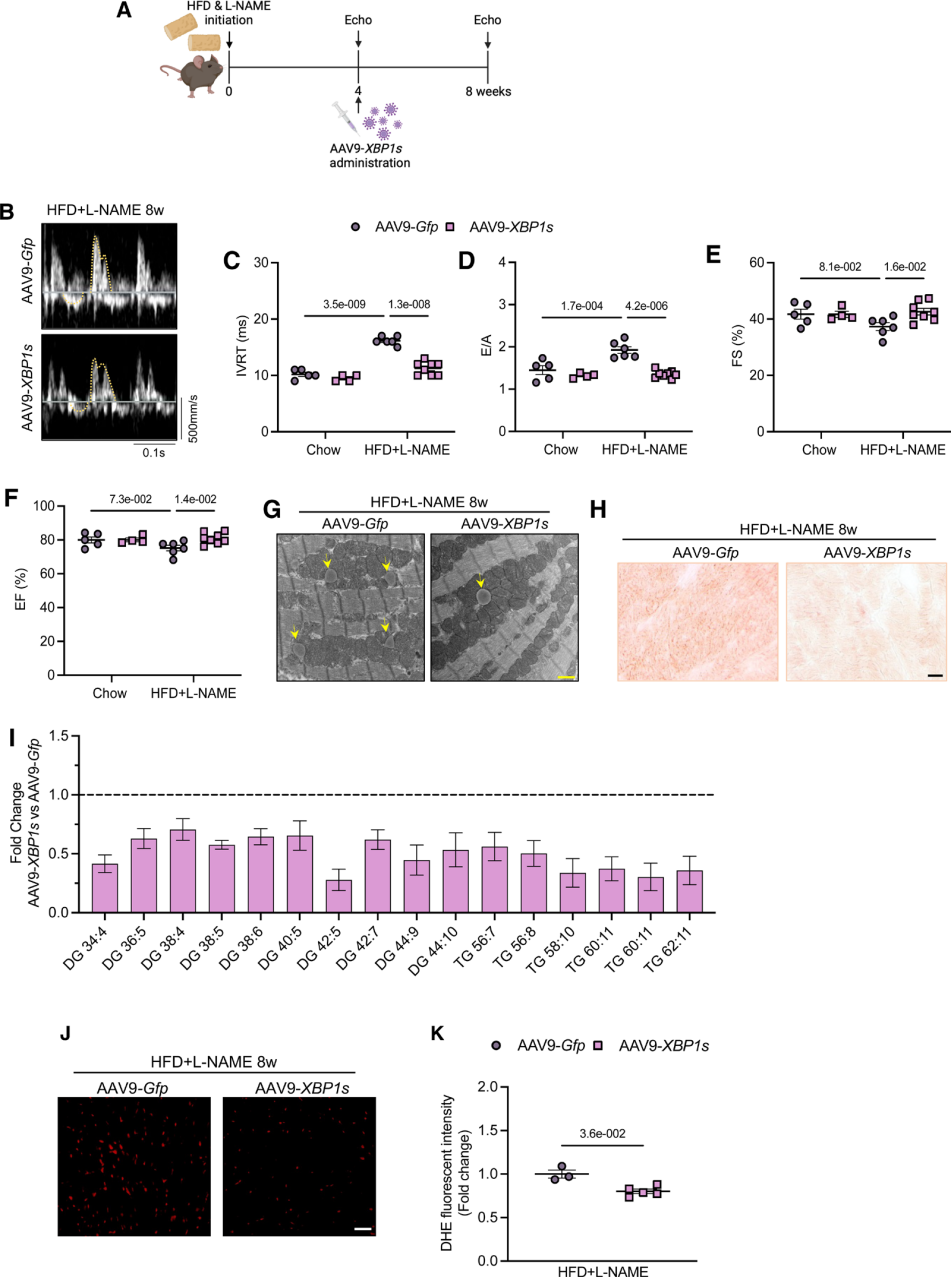
**Spliced X-box binding protein 1 overexpression ameliorates cardiac lipotoxicity upon metabolic stress. A**, Schematic of the experimental design. **B**, Representative pulsed-wave Doppler tracings. **C** and **D**, Isovolumic relaxation time (**C**) and ratio of peak velocity blood flow in early diastole to late diastole (**D**) obtained from pulsed-wave Doppler. **E** and **F**, Percentage of left ventricular fractional shortening (**E**) and percentage of ejection fraction (**F**) obtained from M-mode echocardiography (n=4–8 mice). **G**, Representative transmission electron microscopy images (arrows indicate lipid droplets; scale bar=1 µm). **H**, Oil red O staining (scale bar=20 µm). **I**, Lipidomics showing significantly changed diglycerides and triglycerides in hearts overexpressing XBP1s (spliced X-box binding protein 1) compared with control hearts under metabolic stress induced by a high-fat diet and Nω-nitro-L-arginine methyl ester (n=6 hearts, *P*<0.05). **J**, Representative images of dihydroethidium staining (scale bar=50 µm). **K**, Quantification of dihydroethidium intensity (n=3–5 hearts). Data are presented as mean±SEM *P* values were calculated using a 2-way ANOVA with Šidák post hoc tests (**C** to **F**), an unpaired Student *t* test (**I**), or a Mann-Whitney test (**K**). DG indicates diglyceride; DHE, dihydroethidium; E/A, ratio of peak velocity blood flow in early diastole to late diastole; EF%, percentage of ejection fraction; FS%, percentage of left ventricular fractional shortening; HFD, high-fat diet; IVRT, isovolumic relaxation time; L-NAME, Nω-nitro-L-arginine methyl ester; and TG, triglyceride.

Second, we affirmed the treatment potential of maintaining cardiac XBP1s in preventing HFpEF. Mice injected with AAV9-*XBP1s* were subject to HFpEF-mimicking stress (Figure 7A), which also showed a maintained myocardial EDEM2 (Figure S24A and S24B). Preserved cardiac function was observed by cardiac XBP1s restoration, regardless of metabolic changes (Figure S24C and S24D; Figure 7B through 7F; Table S6). Metabolic stress-triggered LD accumulation, higher levels of DGs and TGs, and oxidative stress accompanied by hypertrophic growth were prevented by reinstatement of XBP1s in the myocardium (Figure 7G through 7K; Figure S24E). All data verify that XBP1s is cardioprotective via EDEM2 against HFpEF.

### The XBP1s-EDEM2 Axis Confers Lipid Homeostasis in Human Induced Pluripotent Stem Cell–Derived Cardiomyocytes

**Figure 8.**
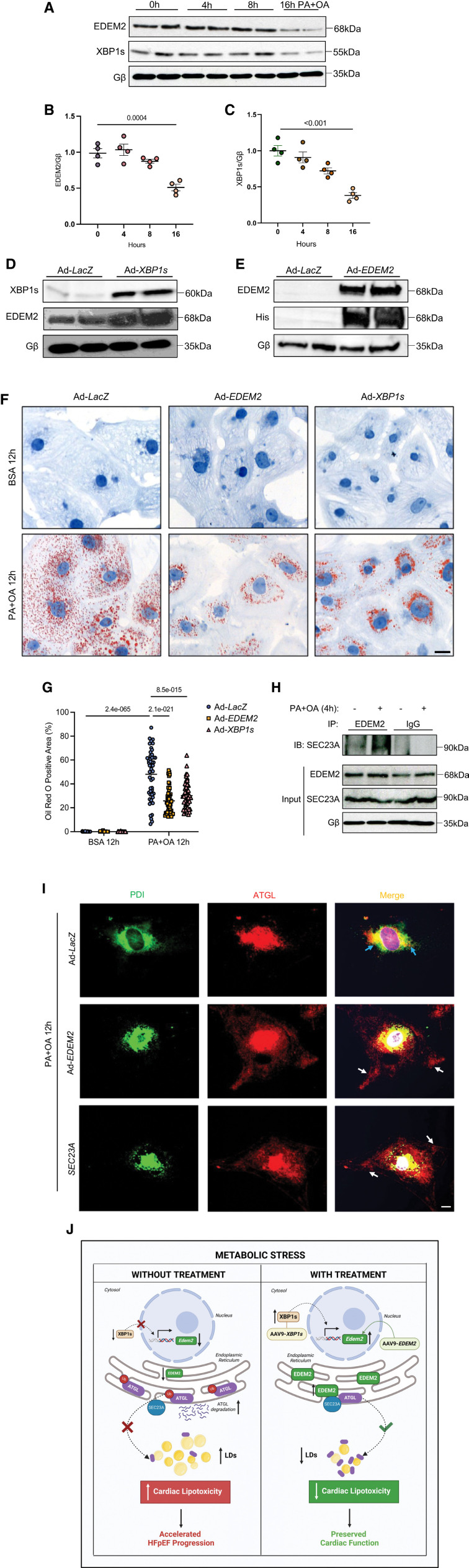
**The s**pliced X-box binding protein 1 **and endoplasmic reticulum degradation–enhancing alpha-mannosidase–like protein 2 pathway is required to facilitate adipose triglyceride lipase translocation to inhibit excessive lipid droplets in human induced pluripotent stem cell–derived cardiomyocytes. A**, Representative immunoblots of EDEM2 (endoplasmic reticulum degradation–enhancing alpha-mannosidase–like protein 2) and XBP1s (spliced X-box binding protein 1) in response to stimulation of palmitic acid and oleic acid (300 μM each). **B** and **C**, Quantification of EDEM2 (**B**) and quantification of XBP1s (**C**) in human induced pluripotent stem cell–derived cardiomyocytes (n=4 experiments). **D**, Immunoblots showing XBP1s overexpression and higher EDEM2 expression in human induced pluripotent stem cell–derived cardiomyocytes. **E**, Immunoblots validating EDEM2 overexpression. **F**, Representative oil red O staining images (scale bar=20 µm). **G**, percentage of oil red O–positive area (n=50 cells in 3 experiments). **H**, Co-immunoprecipitation demonstrating the association of endogenous EDEM2 and SEC23A upon acute stress. **I**, Longer-term stress resulted in endoplasmic reticulum–retained ATGL (adipose triglyceride lipase; red) (PDI [protein disulfide isomerase; green] is an endoplasmic reticulum marker marker), which was diminished by either SEC23A or EDEM2 overexpression. Nuclei were stained with 4',6-diamidino-2-phenylindole (DAPI; blue). White arrows indicate cytosolic translocated ATGL, whereas blue arrows indicate endoplasmic reticulum–retained ATGL (scale bar=20 µm). Data are presented as mean±SEM. *P* values were calculated using a 1-way ANOVA and Tukey post hoc tests (**B** and **C**) or 2-way ANOVA with Šidák post hoc tests (**G**). **J**, Schematic model summarizing the molecular picture of XBP1s-EDEM2 governing lipid homeostasis. XBP1s regulation of EDEM2 facilitates SEC23A-mediated ATGL translocation and inhibits ATGL degradation, mitigating myocardial lipotoxicity and heart failure with preserved ejection fraction under metabolic stress (BioRender). HFpEF indicates heart failure with preserved ejection fraction; LD, lipid droplet; OA, oleic acid; and PA, palmitic acid.

Finally, we gained human-relevant evidence showing that coordinating XBP1s-EDEM2 inhibits lipid overload in response to metabolic stress. Human induced pluripotent stem cell–derived cardiomyocytes were treated with FAs, whereby the expression of XBP1s and EDEM2 was reduced under prolonged stress (Figure 8A through 8C). Either XBP1s or EDEM2 overexpression (Figure 8D and 8E) alleviated LD accumulation (Figure 8F and 8G). Akin to the previous observations, the association of endogenous EDEM2 and SEC23A was detected under short-term stress (Figure 8H). This supports the assumption that the interaction of EDEM2 and SEC23A is evoked to counteract the lipid disturbance insult. As anticipated, overexpression of either EDEM2 or SEC23A deterred ER retention of ATGL upon long-term stress (Figure 8I). Therefore, the data advocate the theory that XBP1s-EDEM2 is required for accurate SEC23A-mediated localization of ATGL, preventing excessive lipids in cardiomyocytes (Figure 8J).

## DISCUSSION

We reveal that cardiac lipotoxicity is a prominent inducer of HFpEF. Impaired lipid equilibrium contributes to disruption of the XBP1s-EDEM2 pathway. Reinstating XBP1s-EDEM2 ameliorates cardiac steatosis and dysfunction. These findings illustrate the molecular basis underlying myocardial lipid overload and provide new therapeutic strategies.

### XBP1s Regulation of EDEM2 in HFpEF Hearts

HFpEF is a predominant cardiac phenotype in metabolic disorders, which can precede the development of HF with reduced ejection fraction. The ER governs modification and targeted transportation of the cellular proteome, including proteins embedded in membranes, retained in cellular compartments, or released extracellularly. We demonstrate that dysregulation of ER-related pathways is a contributor to myocardial lipid overload, which worsens cardiac function. Specifically, we detected a reduction in *XBP1* in HFpEF hearts, consistent with previous findings,^25^ and further identified EDEM2 as a novel transcriptional target of XBP1s in cardiomyocytes. Furthermore, using a preclinical HFpEF model,^25^ XBP1s or EDEM2 deficiency resulted in cardiac diastolic dysfunction and accelerated systolic dysfunction with protruding myocardial lipid accumulation. This indicates that lipid disturbance is causatively involved in the pathogenesis of HFpEF and early transition to HF with reduced ejection fraction. Conversely, EDEM2 overexpression alleviated the deleterious effects, shielding the heart from metabolic disorder–induced insult, particularly in XBP1s deficiency–induced HFpEF hearts. Last, adding to previous observations that XBP1s overexpression preserves cardiac performance,^2^ we showed that heart-specific XBP1s restoration treated HFpEF, providing tangible evidence of the therapeutic potential of targeting this pathway.

### The Indispensable Role of EDEM2 in Preventing Cardiac Lipotoxicity

EDEM2 can regulate protein glycosylation and degradation^18^ and is involved in cellular homeostasis.^19^ For instance, a heterozygous EDEM2 variant causes monogenic childhood-onset diabetes, highlighting it as a diabetes-related gene.^22^ Although its role in protein modification will be investigated, the present observation of colocalization of EDEM2 and ERGIC53 unveils a noncanonical function of EDEM2 for protein trafficking. More importantly, our study provides new insight into the mechanism underlying regulation of cardiac lipid metabolism. We detected the presence of toxic lipid species in human metabolic stress–associated hearts and mouse HFpEF hearts, including long-chain FA–containing DGs and TGs (C18, C20, and C24), contributing to oxidative stress and myocardial pathological remodeling.^32^ Accordingly, EDEM2 knockdown mice subjected to metabolic stress exhibited augmented hypertrophy and oxidative stress. On the contrary, EDEM2 restoration attenuated these effects. The fact that the modulators of intracellular lipid homeostasis, Alda-1 and oxytocin,^28,29^ rescued EDEM2 knockdown–induced cardiac dysfunction supports the theory that EDEM2 loss triggers HFpEF at least partially by damaging cardiac lipid balance. However, further studies are required to explore the roles of specific species of lipids in HF.

TGs are synthesized by ER-resident enzymes that localize to LDs, augmenting the TG amount. We observed a negative correlation between EDEM2 and TG content in cardiomyocytes, in line with the finding that ER-related factors can negatively regulate TG synthesis.^33^ However, the lipogenic enzyme diacylglycerol O-acyltransferase 2 did not exhibit a significant change related to EDEM2, likely excluding the impact of EDEM2 on direct TG synthesis. On the other hand, elevated lipid uptake promotes lipid overload, which was evidenced by the fact that lipid accumulation and reduced cardiac function are associated with increased CD36 and FA transport proteins.^34^ However, EDEM2 reduction or overexpression did not have significant impacts on CD36 protein, indicating comparable lipid uptake regardless of EDEM2.

### EDEM2 Regulation of ATGL in Cardiomyocytes

ATGL is the rate-limiting lipase involved in lipid hydrolysis. ATGL deficiency in mice causes excessive cardiac lipid accumulation and cardiac dysfunction by influencing lipid-associated genes.^6^ Conversely, cardiomyocyte-specific ATGL overexpression leads to a significant reduction in myocardial TG content and sustained cardiac function in response to various pathological stresses.^13,14,35^ In light of unchanged ATGL transcripts regardless of EDEM2 expression, it was speculated that EDEM2 affects ATGL through a posttranscriptional manner, as evidenced by the inhibition of ATGL delivery to LDs under EDEM2 disruption conditions. This is consistent with observations in other cell types where disruptions in protein trafficking pathways strengthen the distribution of ATGL in the ER.^15,16,23^ In addition, our study indicates that ER-retained ATGL triggers its degradation and dysfunction.

Here, we acknowledge a novel regulatory mechanism of ATGL in cardiomyocytes. Pull-down proteomics detected the interaction between EDEM2 and SEC23A. SEC23A is a mediator that physically deforms the ER membrane into vesicles and selects protein cargos.^36^ Notably, the endogenous interaction between EDEM2 and SEC23A was enhanced under acute stimulation of FAs, where COPII trafficking increases,^37^ which explains why ER retention of ATGL in cardiomyocytes occurred via EDEM2 or SEC23A knockdown. Ultimately, ER-restrained ATGL tends to be degraded by the proteasome, as evidenced by higher ubiquitinated ATGL attributable to EDEM2 deficit. Moreover, we demonstrate that EDEM2 was positively correlated with ATGL activity; therefore, metabolic stress-induced EDEM2 disruption exasperated cardiac lipotoxicity and the progression of cardiomyopathy, at least partially, via impaired ATGL translocation in cardiomyocytes. However, whether ATGL is tethered to SEC23A-mediated vesicles trafficking to or directly transported to the LDs originating from the ER is unexplored in the current study. Canonically, the association between EDEM2 and the cytoplasmic protein SEC23A could involve additional components; therefore, their interaction and specific cargos in cardiomyocytes also need further investigation.

Myocardial ATGL-mediated lipolysis facilitates lipid turnover and serves as a continuous fuel source.^6,14,35^ Compromised cardiac lipolysis, attributable to ATGL loss of function, causes profound cardiac steatosis, formation of harmful lipid species, and mitochondrial FA oxidation (FAO) insufficiency.^38^ The effects of appropriate ATGL-involved lipolysis were substantiated by our findings, where EDEM2 overexpression reduced TGs and DGs. Likewise, because ABHD5 (CGI-58) is a regulator of lipid hydrolysis by coactivating ATGL, knockdown of hepatic CGI-58 leads to a profound increase in both TG and DG contents in the liver, indicating inadequate lipolysis.^39^ Interestingly, EDEM2 knockdown-induced cardiac lipotoxicity was not ameliorated by an ABHD5 ligand, SR-4995.^40^ The cardiac characteristics unique to EDEM2 deficiency cannot be compensated by the ATGL activator, affirming that EDEM2 action on lipid metabolism is through the regulation of ATGL.

### Impacts of EDEM2 on Mitochondrial FAO

Decreased FAO capacity of the mitochondria also contributes to lipid excess and oxidative stress, acting as a cornerstone of intracellular metabolic disruption.^41,42^ Inadequate FAO aggravates HFpEF during the course of metabolic stress,^27,43,44^ indicating that enhancing FAO serves as a viable strategy to protect the heart from development of HF. Although others have observed increased FAO in HFpEF, the subsequent decline in mitochondrial oxidative phosphorylation leads to defects in cardiac energetics.^45^ Thus, emerging evidence supports the principle that maintaining the balance of cardiac FA turnover and use is ideal to avoid cardiac lipotoxicity and energy deprivation in HFpEF.

Pathway analyses revealed that HFpEF hearts or EDEM2-deficient cells displayed dysregulated genes for lipid handling, the citric acid cycle, and anti-oxidative responses. Specifically, under metabolic stress, EDEM2 positively correlated with genes associated with mitochondrial FAO (*Cpt1b* and *Acadl*) and anti-oxidative responses (*Cat*). Moreover, loss of EDEM2 or ATGL or blockage of ATGL trafficking reduced ATP in cells. Conversely, long-term FA-lowered ATP concentration was reversed by maintenance of either EDEM2 or XBP1s. Of note, EDEM2 deficiency–reduced ATP was akin to the phenotype of treatment with the CPT1 inhibitor etomoxir. CPT1B is a rate-limiting transporter of FAs into the mitochondria in adult cardiomyocytes. CPT1B deficiency causes lipotoxicity coupled with compromised mitochondrial biogenesis and FAO under pressure overload.^46^ Given that ATGL loss also impairs CPT1B expression,^6^ similar to the features of EDEM2 knockdown, the mechanisms by which EDEM2 regulates mitochondrial FAO need to be deciphered in depth.

### Beneficial Effects of Preventing Cardiac Lipotoxicity

Alda-1, an activator of aldehyde dehydrogenase 2, restores mitochondrial function and FA use, ameliorating lipotoxic cardiomyopathy.^47^ We observed that Alda-1 administration ameliorated lipotoxicity, with favorable outcomes for HFpEF in animal models. As an alternative, oxytocin inhibits the manifestations of overt cardiomyopathy.^48^ Consistently, oxytocin prevented cardiac lipotoxicity and improved cardiac function in the present study. The molecular basis underlying the protective role of oxytocin in the heart through alleviation of cardiac lipotoxicity has not yet been investigated. However, its documented role in enhancing β‐oxidation and lipolysis in adipose tissue may also be applicable in the setting of the heart.^29^ Of note, their beneficial effects under conditions of EDEM2 loss indicates their protective roles in an EDEM2-independent manner; however, the precise mechanisms need to be studied. Nevertheless, these findings not only suggest that dysfunctional lipid metabolism in the myocardium independently worsens HFpEF or aggravates HF with reduced ejection fraction under metabolic stress but also provide proof-of-concept evidence that HF can be prevented or treated by mitigating cardiac lipotoxicity.

### Conclusions

Our data reveal a vital role of cardiac XBP1s-EDEM2 governing lipid homeostasis in the myocardium through modulation of ATGL. Mitigation of cardiac lipotoxicity by targeting this pathway is a promising approach for deferring HFpEF onset and progression.

## ARTICLE INFORMATION

### Acknowledgments

The authors are grateful to several individuals at The University of Manchester for providing valuable assistance throughout this project. First, they express their gratitude to Roger Meadows, Steven Marsden, Peter March, and Darren Thomson of the Bioimaging Facility for technical training on imaging. Additionally, the authors thank Samantha Forbes and Aleksandr Mironov at the Electron Microscopy Core Facility for TEM technical training and support. They also extend their appreciation to Bharatkumar Rash, Claire Morrisroe, Andy Hayes, Ian Donaldson, Rachel Scholey, and Leo Zeff of the Genomic Technologies Facility and Bioinformatics Facility for technical support with RNA sequencing and data analysis. The authors further acknowledge George Taylor, Emma-Jayne Keevil, Julian Selley, Stacey Warwood, Ronan O’Cualain, and David Knight of the Mass Spectrometry Facility for professional advice, processing samples, conducting experiments, and analyzing proteomics and lipidomics data. Finally, they wish to recognize technical support provided by Daniel Byrne of the University of Manchester while using RStudio. W.L. conceptualized the idea for the project detailed in this manuscript. O.F. and W.L. contributed significantly to the experimental design; the majority of animal work; functional and molecular studies; data acquisition, analysis, and interpretation; and manuscript drafting and revision. R.R. conducted some animal work and molecular assessments with help from J.Z., A.R.-V., N.K., Z.Z., and X.C. C.R. and S.R.G. helped with data interpretation. J.M.M., R.R.E.A., Q.O., and T.M.A.M. cultured human heart slices. S.S.H., D.F., and O.J.M. prepared key AAVs. S.M., D.T, C.S.-F., and G.G.S. provided essential materials. E.S. and M.R.P. provided advice on the project. K.K., X.Z., T.W., and L.V. provided technical support for hiPSC experiments. C.P. provided support for TEM experiments. E.J.C. reviewed in vivo studies. This manuscript was primarily drafted by W.L., with contributions from O.F., C.R., and S.R.G. M.K.R., and B.D.K. provided mentorship and reviewed the manuscript. B.D.K. was supported by a British Heart Foundation personal chair.

### Sources of Funding

This work was supported by the British Heart Foundation (FS/15/16/31477, FS/18/73/33973, FS/19/70/34650, PG/19/66/34600, FS/PhD/22/29307, PG/22/10904, and PG/22/11075 to W.L.; BHF accelerator award AA/18/4/34221 to the University of Manchester; FS/18/4/33310 to C.P.; and PG/23/11662 to L.V.), a professorship from the German Center for Cardiovascular Research (81Z0700201 to O.J.M.), and grants from the DZHK (German Center for Cardiovascular Research) to G.G.S. and the Deutsche Forschungsgemeinschaft (DFG; German Research Foundation-SFB-1470-A02 to G.G.S.). T.M.A.M. is supported by National Institutes of Health grants R01HL147921 and P30GM127607, Department of Defense grant W81XWH-20-1-0419, and American Heart Association grant 16SDG29950012. The authors also acknowledge National Institutes of Health grant F32HL149140 (to R.R.E.A). This work was also supported by the NIHR Manchester Biomedical Research Centre (NIHR203308).

### Disclosures

T.M.A.M. holds equities at Tenaya Therapeutics.

### Supplemental Material

Expanded Methods

Figures S1–24

Tables S1–S6

References 49–64
